# Experimental and Theoretical Investigation on the Thermochemistry of 3-Methyl-2-benzoxazolinone and 6-Nitro-2-benzoxazolinone

**DOI:** 10.3390/molecules27010024

**Published:** 2021-12-21

**Authors:** Ana L. R. Silva, Vânia M. S. Costa, Maria D. M. C. Ribeiro da Silva

**Affiliations:** Centro de Investigação em Química (CIQUP), Department of Chemistry and Biochemistry, Faculty of Sciences, University of Porto, Rua do Campo Alegre, 687, P-4169-007 Porto, Portugal; vaniasilvacosta@gmail.com (V.M.S.C.); mdsilva@fc.up.pt (M.D.M.C.R.d.S.)

**Keywords:** benzoxazolone, formation enthalpy, sublimation enthalpy, Gibbs enthalpy of formation, enthalpic increments, high-level calculations

## Abstract

The determination of the reliable thermodynamic properties of 2-benzoxazolinone derivatives is the main goal of this work. Some correlations are established between the energetic properties determined and the structural characteristics of the title compounds, and the reactivity of this class of compounds is also evaluated. Static-bomb combustion calorimetry and high-temperature Calvet microcalorimetry were used to determine, respectively, the standard molar enthalpies of formation in the solid state and the standard molar enthalpies of sublimation, both at *T* = 298.15 K. Using the results obtained for each compound, the respective gas-phase standard molar enthalpy of formation was derived. High-level quantum chemical calculations were performed to estimate the same property and the results evidence good accordance. Moreover, the gas-phase relative thermodynamic stability of 2-benzoxazolinone derivatives was also evaluated using the respective gas-phase standard molar Gibbs energy of formation. In addition, the relationship between the energetic and structural characteristics of the benzoxazolinones is presented, evidencing the enthalpic increments associated with the presence of a methyl and a nitro groups in the molecule, and this effect is compared with similar ones in other structurally related compounds.

## 1. Introduction

**Figure 1:**
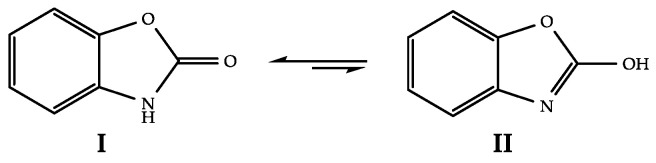
Amide tautomer of 2-benzoxazolinone (**I**) and the corresponding enol form (**II**).

Benzoxazolinone (BOA) is a heterocyclic compound whose structure consists of a benzene ring fused with a five-membered ring containing oxygen and nitrogen as heteroatoms. A possible tautomeric form of BOA is formed as a result of the removal of hydrogen from the nitrogen atom in the ring to the oxygen of the carbonyl group (Figure 1). This enol form of BOA is 2-benzoxazolol (Figure 1, II), energetically much less stable than its amide tautomer by ca. 60 kJ mol^−1^ [1]. 

Compounds presenting the 2-benzoxazolinone (BOA) scaffold have been promising key structures for the design and development of novel drugs [2]. Many studies confirm BOA-based derivatives as having a diverse number of pharmacological activities including anticonvulsant, antipyretic, analgesic, cardiotonic, antiulcer, antibacterial, antimicrobial, and antifungal effects [2]. On the other hand, BOA derivatives, being allelochemicals, have the potential to act as a natural pesticide. Thus, they act as an important factor of resistance of the host plant against microbial diseases and insects [3].

Structural modification within the core scaffold of BOA has been widely explored in positions 3 and 6 by making different substitutions, which contributes to molecules with different structural characteristics and pharmacological properties.

**Figure 2:**
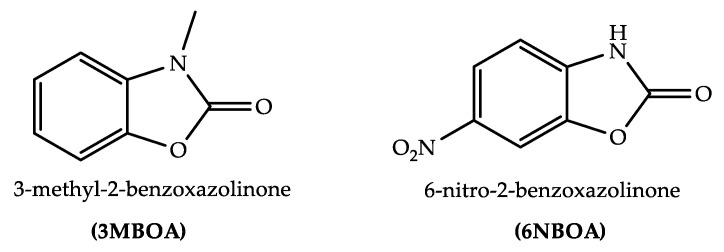
Structural formula of the studied compounds.

It is important to understand the physicochemical characterization of BOA derivatives, as a support for the discovery of new entities with a fundamental role in medicinal chemistry. Our research group was involved in a systematic experimental and computational study on the thermodynamic characterization of nitrogen-containing heterocycles, namely benzoxazoles, benzothiazoles and imidazoles/benzimidazoles [4,5,6,7]. Moreover, Morais et al. carried out a thermodynamic study of2-benzoxazolinone, in addition to2-benzimidazolinone and 3-indazolinone [1]. Other compounds with similar structures were also studied, namely 2,3-dioxoindole [8], oxindole [9], 1,2-benzisothiazol-2(3*H*)-one and 1,4-benzothiazin-3(2*H*,4*H*)-one [10]. Continuing our research interests, in this work, a thermochemical study of two nitrogen-containing heterocycles, 3-methyl-2-benzoxazolinone and 6-nitro-2-benzoxazolinone (Figure 2), was performed experimentally, using combustion calorimetry and microcalorimetry Calvet. In addition, density-functional theory calculations were carried out, allowing the estimation of reaction and formation enthalpies.

## 2. Results and Discussion

### 2.1. Massic Energies of Combustion

The standard (*p*° = 0.1 MPa) massic energies of combustion of the compounds studied were determined using a static-bomb combustion calorimeter. The results of all the combustion experiments and the individual values of the massic energy of combustion, Δ_c_*u*°, with the mean value and the corresponding standard deviation of the mean, for the studied compounds are given in Supplementary Materials (Tables S1 and S2). The internal energy for the isothermal bomb process, Δ*U*(IBP), was calculated by Equation (1), where $\varepsilon_{\mathrm{cal}}$ is the energy equivalent of the calorimeter (see Section 3.2), $\varepsilon_{f}\;$is the energy equivalent of the contents in the final state, Δ*T*_ad_ is the corrected temperature rise obtained in each experiment, and Δ*U*(ign) is the electrical energy for ignition.

(1) $$\Delta U(\mathrm{IBP})=-\left(\varepsilon_{\mathrm{cal}}+\varepsilon_{f}\right)\Delta T_{ad}+\Delta U(\mathrm{ign})$$

The values of the standard massic energies of combustion, Δ_c_*u*°, for 3MBOA and 6NBOA are referred to the combustion reactions (2) and (3), respectively. Table 1 lists, for both compounds, the values of the standard massic energy of combustion, Δ_c_*u*°(cr), of the derived standard molar energy and enthalpy of combustion, $\Delta_{c}U_{m}^{{}^{\circ}}\left(\mathrm{cr}\right)$ and $\Delta_{c}H_{m}^{{}^{\circ}}\left(\mathrm{cr}\right)$, as well as of the standard molar enthalpy of formation, in the condensed phase, $\Delta_{f}H_{m}^{{}^{\circ}}\left(\mathrm{cr}\right)$, at *T* = 298.15 K. The uncertainty associated with each value of the standard molar enthalpy of combustion is twice the overall standard deviation of the mean and includes the uncertainties inherent in the calibration constant and in the values of the combustion energies of the auxiliary materials [11,12]. To derive $\Delta_{f}H_{m}^{{}^{\circ}}\left(\mathrm{cr}\right)$ from $\Delta_{c}H_{m}^{{}^{\circ}}\left(\mathrm{cr}\right),\;$the standard molar enthalpies of formation for H_2_O(l): –(285.830 ± 0.040) $\mathrm{kJ}\cdot {\mathrm{mol}}^{-1}$ and for CO_2_(g): –(393.51 ± 0.13) $\mathrm{kJ}\cdot {\mathrm{mol}}^{-1}$, were used [13].

C_8_H_7_NO_2_(cr) + 8.75O_2_(g) → 8CO_2_(g) + 3.5H_2_O(l) + 0.5N_2_(g) (2)

C_7_H_4_N_2_O_4_(cr) + 6O_2_(g) → 7CO_2_(g) + 2H_2_O(l) + N_2_(g) (3)

### 2.2. Enthalpies of Sublimation

The standard molar enthalpies of sublimation, $\Delta_{\mathrm{cr}}^{g}H_{m}^{{}^{\circ}}$, for the studied compounds were determined by Calvet microcalorimetry. Table 2 presents the results of these experiments. The total enthalpy change for each experiment, at the experimental temperature *T*, $\Delta_{\mathrm{cr},\;298.15\;K}^{g,\;T}H_{m}^{{}^{\circ}}$, was converted to *T* = 298.15 K, $\Delta_{\mathrm{cr}}^{g}H_{m}^{{}^{\circ}},\;$through Equation (4), using the enthalpic term $\Delta_{298.15\;K}^{T}H_{m}^{{}^{\circ}}\left(g\right).\;$This last parameter is calculated using the gas-phase molar heat capacities, $C_{p,\;m\;}^{{}^{\circ}}$(g), derived from statistical thermodynamics, using the vibrational frequencies obtained at B3LYP/6-31G(d) level [14], scaled by a factor of 0.9613 [15]. The values of $C_{p,\;m\;}^{{}^{\circ}}$(g) = ƒ(*T*), listed in Tables S3 and S4, are well adjusted by third-degree polynomials (more information in ESI).

(4) $$\Delta_{298.15\;K}^{T}H_{m}^{{}^{\circ}}(g)=\int_{298.15\;K}^{T}C_{p,m}^{{}^{\circ}}(g)\;dT$$

### 2.3. Enthalpies of Formation in the Gaseous Phase

Combining the results of the standard molar enthalpies of formation, in the crystalline phase, and the standard molar enthalpies of sublimation, we calculated the standard molar enthalpies of formation in the gaseous phase, $\Delta_{f}H_{m}^{{}^{\circ}}\left(g\right)$ at *T* = 298.15 K, for the studied compounds. All values are summarized in Table 3. This property was also calculated by a computational method, the G3MP2B3 composite method, widely tested in our research group, with reliable results for this type of heterocycle [5,16]. The working reactions used to estimate the enthalpies of formation of 3MBOA and 6NBOA are collected in Tables S5 and S6, respectively. In Table 4, the experimental and estimated values of $\Delta_{f}H_{m}^{{}^{\circ}}\left(g\right),\;$at *T* = 298.15 K, are compared for the two benzoxazolinones studied. Analysing the results, it is possible to confirm the excellent agreement between experiment and theory. The maximum deviation between DFT estimates and experimental enthalpies of formation is 4.1 $\mathrm{kJ}\cdot {\mathrm{mol}}^{-1}$.

**Scheme 1:**
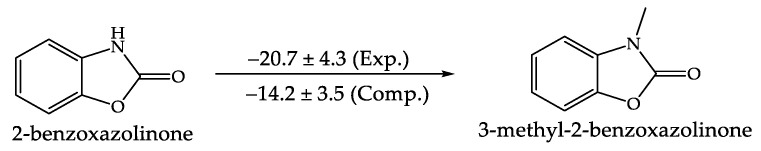
Experimental and computational enthalpic increments from 2-benzoxazolinone to 3-methyl-2-benzoxazolinone (values in $\mathrm{kJ}\cdot {\mathrm{mol}}^{-1}$).

**Scheme 2:**
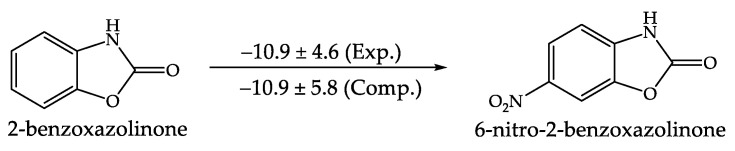
Experimental and computational enthalpic increments from 2-benzoxazolinone to 6-nitro-2-benzoxazolinone (values in $\mathrm{kJ}\cdot {\mathrm{mol}}^{-1}$).

Moreover, we calculated the experimental and computational enthalpic increments from 2-benzoxazolinone to 3-methyl-2-benzoxazolinone and 6-nitro-2-benzoxazolinone, Scheme 1 and Scheme 2, respectively, using the data from Table 4. The insertion of one methyl or nitro group in the benzoxazolinone core is enthalpically favourable and, again, the calculated and the experimental enthalpic increments are in good agreement, considering the associated uncertainties. Considering the results of $\Delta_{f}{H\;}_{m}^{\;{}^{\circ}}\left(g\right)$ in the literature for molecules with similar structures, namely 2-pyrrolidinone [17], 1-methyl-2-pyrrolidinone [17], 2,3-pyrrolidinedione [18], 1-methyl-2,3-pyrrolidinedione [19], 2,3-dioxoindole [17] and 1-methyl-2,3-dioxoindole [17], we calculated the enthalpic increments for the substitution of the hydrogen atom of the N-heteroatom of the pentagonal ring by a methyl group. The aforementioned energetic substitutions are reflected into the following enthalpic increments: 2-pyrrolidinone → 1-methyl-2-pyrrolidinone (−13.4 ± 3.2 $\mathrm{kJ}\cdot {\mathrm{mol}}^{-1}$), 2,3-pyrrolidinedione → 1-methyl-2,3-pyrrolidinedione (−14.2 ± 2.2 $\mathrm{kJ}\cdot {\mathrm{mol}}^{-1}$) and 2,3-dioxoindole → 1-methyl-2,3-dioxoindole (−15.6 ± 7.1 $\mathrm{kJ}\cdot {\mathrm{mol}}^{-1}$). These results compare very well with those obtained in this work (Scheme 1). Concerning the substitution of the hydrogen atom in the benzenic ring of BOA by a nitro group, we obtained a negative value for the respective enthalpic increment, (≈−11 $\mathrm{kJ}\cdot {\mathrm{mol}}^{-1}$, Scheme 2), which is in good agreement with one study developed by us for benzoxazole and benzothiazole [4]. However, a different finding was obtained in the study of Ribeiro da Silva et al. [20], in which identical substitution in 2,1,3-benzothiadiazole, benzofurazan and benzofurazan-1-oxide is enthalpically not favourable, i.e., slightly positive. In these cases, the nitro group is closer to the N-heteroatom of the pentagonal ring, which may affect this analysis. 

### 2.4. Thermodynamic Stability of Compounds in the Gaseous Phase

The relative thermodynamic stability of benzoxazolinones in the gaseous phase can be evaluated knowing the corresponding standard molar Gibbs energies of formation,$\;\Delta_{f\;}G_{m}^{{}^{\circ}}(g),$ derived from Equation (5).

(5) $$\Delta_{f}G_{m}^{{}^{\circ}}{(g)=\Delta }_{f}H_{m}^{{}^{\circ}}\;\left(g\right)-{T\Delta }_{f}S_{m}^{{}^{\circ}}\left(g\right)$$

For these calculations, the following standard reference entropy values were used [21]: $S_{m}^{{}^{\circ}}$(C, graphite) = 5.740 J·K^−1^·mol^−1^, $S_{m}^{{}^{\circ}}$(H_2_, g) = 130.680 J·K^−1^·mol^−1^, $S_{m}^{{}^{\circ}}$(N_2_, g) = 191.609 J·K^−1^·mol^−1^ and $S_{m}^{{}^{\circ}}$(O_2_, g) = 205.147 J·K^−1^·mol^−1^, as well as the values of${\;S}_{m}^{{}^{\circ}}$(g) of each compound calculated at the B3LYP/6-31G(d) level of theory, using a vibrational frequency scaling factor of 1.0029 [15]. In Supplementary Information the harmonic frequencies calculated for the gaseous molecules are presented. Table 5 collects the${\;S}_{m}^{{}^{\circ}}$(g) of each compound and the standard molar enthalpies and Gibbs energies of formation in gaseous phase. 

Analysing these results, it is possible to conclude that the stability respects the following tendency, BOA > 3MBOA > 6NBOA, at standard conditions and at *T* = 298.15 K, in the gaseous phase. As can be observed, the decreasing order of the values of the Gibbs energy of formation from 6NBOA to BOA differs from that observed for the enthalpy of formation. This is mainly due to the differences in the entropies of formation. BOA is the most stable of the three, considering that its entropic contribution is the strongest.

## 3. Materials and Methods

### 3.1. Materials

The commercially obtained compounds were purified by sublimation under reduced pressure. Details about the initial and final purity grades of each compound are presented in Table 6. The purity analyses were carried out on an Agilent 4890D gas chromatography–flame ionization detector (GC–FID) apparatus, equipped with an HP-5 column.

The standard atomic masses recommended by the IUPAC Commission in 2011 were used in the calculation of all molar thermodynamic quantities [22].

### 3.2. Combustion Calorimetry

The bomb used was a twin-valve bomb, the internal volume of which was 0.342 dm^3^ (type 1108 of Parr Instrument Company). The apparatus and technique were described previously [23,24]. The calibration of the calorimeter was performed by the combustion of benzoic acid Thermochemical Standard, sample NBS 39j, with massic energy of combustion, under bomb conditions, of –(26434 ± 3) J⋅g^–1^, and corrected to give the energy equivalent, $\varepsilon_{\mathrm{cal}}$, corresponding to the average mass of water added to the calorimeter (3119.6 g). The energy equivalent of the calorimeter was determined,${\;\varepsilon }_{\mathrm{cal}}\;$= (16,002.6 ± 1.7) J⋅K^−1^, where the uncertainty quoted is the standard deviation of the mean.

The standard massic energy of combustion, $\Delta_{c}u^{{}^{\circ}}$, was calculated by a similar procedure to that developed by Hubbard et al. [25]. Further details about the experimental procedure are provided in ESI.

### 3.3. High-Temperature Microcalorimetry

The enthalpies of sublimation of the studied compounds were measured with a high-temperature Calvet microcalorimeter (Setaram HT 1000), using the “vacuum-sublimation”-drop microcalorimetric method described by Skinner [26]. The details of the apparatus and the technique used were previously described in detail [27].

Samples of about 5 to 10 mg of the compounds, contained in thin-glass capillary tubes, were simultaneously dropped at room temperature into each of the calorimetric cells (reference and reaction) held at *T* = 361 K for 3MBOA and *T* = 483 K for 6NBOA, and then removed from the hot zone by vacuum sublimation. A correction, *k*, to the internal calibration constant of the calorimeter was obtained for each temperature: for *T* = 361 K, *k* = (1.044 ± 0.008), as the average of six independent experiments with naphthalene; for *T* = 483 K, *k* = (1.071 ± 0.008), corresponding to the average of six independent experiments with anthracene.

### 3.4. Computational Details

The standard ab initio molecular orbital calculations for the compounds studied were performed using the composite G3(MP2)//B3LYP method [28]. The geometries and zero-point energies are obtained from the B3LYP density-functional theory (B3LYP/6-31G(d)) and the single-point calculations are carried out at higher levels of electronic-structure theory. The absolute enthalpies of the molecules studied, at 298.15 K, were obtained by adding the energies computed at 0 K with the vibrational, translational, rotational contributions and the *RT* terms. All standard computational calculations were performed using the Gaussian 09 software package [29].

## 4. Conclusions

This experimental thermochemical study enabled the determination of new values for the enthalpy of formation, in the crystalline phase, and for the enthalpy of sublimation of two benzoxazolinones. In addition, new values for the gas-phase enthalpy of formation were derived and compared with the estimates by the G3MP2B3 approach. The experimental and computational enthalpic increments were derived: 2-benzoxazolinone → 3-methyl-2-benzoxazolinone (mean value: −16.8 ± 2.7 $\mathrm{kJ}\cdot {\mathrm{mol}}^{-1}$) and 2-benzoxazolinone → 6-nitro-2-benzoxazolinone (mean value: −10.9 ± 3.6 $\mathrm{kJ}\cdot {\mathrm{mol}}^{-1}$). These results reveal that the insertion of one methyl or nitro group in the benzoxazolinone core is enthalpically favourable. 

In relation to the relative thermodynamic stability of the compounds, it was shown that 2-benzoxazolinone (BOA) is the most stable in the gaseous phase. 

## Figures and Tables

**Table 1:** Standard (*p°* = 0.1 MPa) massic energy of combustion, $\Delta_{c}u^{{}^{\circ}},$ molar energy of combustion,$\;\Delta_{c}U_{m}^{{}^{\circ}},\;$enthalpy of combustion, $\Delta_{c}H_{m}^{{}^{\circ}},\;$and enthalpy of formation, $\Delta_{f}H_{m}^{{}^{\circ}},\;$for the studied compounds, at *T* = 298.15 K. ^1^

Compound	$\Delta_{c}u^{{}^{\circ}}(\mathrm{cr})/\;J\cdot g^{-1}$	$\Delta_{c}U_{m}^{{}^{\circ}}\left(\mathrm{cr}\right)/\;\mathrm{kJ}\cdot {\mathrm{mol}}^{-1}$	$\Delta_{c}H_{m}^{{}^{\circ}}\left(\mathrm{cr}\right)/\;\mathrm{kJ}\cdot {\mathrm{mol}}^{-1}$	$\Delta_{f}H_{m}^{{}^{\circ}}\left(\mathrm{cr}\right)/\;\mathrm{kJ}\cdot {\mathrm{mol}}^{-1}$
3MBOA	−25,637.5 ± 6.9	−3823.7 ± 2.2	−3824.4 ± 2.2	−324.1 ± 2.5
6NBOA	−16,484.0 ± 3.4	−2969.1 ± 1.5	−2964.1 ± 1.5	−362.1 ± 1.7

Abbreviations: 3MBOA, 3-methyl-2-benzoxazolinone; 6NBOA, 6-nitro-2-benzoxazolinone. ^1^ Uncertainties are twice the overall standard deviation of the mean, and include the contributions from the calibration with benzoic acid.

**Table 2:** Standard (*p*° = 0.1 MPa) molar enthalpies of sublimation, $\Delta_{\mathrm{cr}}^{g}H_{m}^{{}^{\circ}}$, at *T* = 298.15 K, determined by Calvet microcalorimetry for the compounds studied.

Compound	*T*_exp_^1^/K	$\Delta_{\mathrm{cr},\;298.15\;K}^{g,\;T}H_{m}^{{}^{\circ}}{\;}^{1}/\mathrm{kJ}\cdot {\mathrm{mol}}^{-1}$	$\Delta_{298.15\;K}^{T}H_{m}^{{}^{\circ}}\left(g\right){\;}^{2}/\mathrm{kJ}\cdot {\mathrm{mol}}^{-1}$	$\Delta_{\mathrm{cr}}^{g}H_{m}^{{}^{\circ}}{\;}^{3}/\mathrm{kJ}\cdot {\mathrm{mol}}^{-1}$
3MBOA	360.51 ± 0.03	99.77 ± 0.28	10.38 ± 0.01	89.4 ± 2.1
6NBOA	482.92 ± 0.05	170.25 ± 0.63	38.01 ± 0.01	132.2 ± 3.3

Abbreviations: 3MBOA, 3-methyl-2-benzoxazolinone; 6NBOA, 6-nitro-2-benzoxazolinone. ^1^ Uncertainties are the standard deviation of the mean of six independent experiments. ^2^ Uncertainties calculated through the RSS method. ^3^ Uncertainties are the expanded uncertainties of the mean (0.95 level of confidence, *k* = 2) and include the uncertainties due to the calibration.

**Table 3:** Experimentally derived standard (*p°* = 0.1 MPa) molar enthalpies of formation, in the gaseous phase, at *T* = 298.15 K, for the compounds studied.

Compound	$\;\Delta_{f\;}H_{m\;}^{{}^{\circ}}(\mathrm{cr})/\mathrm{kJ}\cdot {\mathrm{mol}}^{-1}\;$	$\;\Delta_{\mathrm{cr}}^{g}H_{m}^{{}^{\circ}}/\mathrm{kJ}\cdot {\mathrm{mol}}^{-1}\;$	$\;\Delta_{f\;}H_{m}^{{}^{\circ}}\left(g\right){\;}^{1}/\mathrm{kJ}\cdot {\mathrm{mol}}^{-1}$
3MBOA	−324.1 ± 2.5	89.4 ± 2.1	−239.7 ± 3.2
6NBOA	−362.1 ± 1.7	132.2 ± 3.3	−229.9 ± 3.7

Abbreviations: 3MBOA, 3-methyl-2-benzoxazolinone; 6NBOA, 6-nitro-2-benzoxazolinone. ^1^ Uncertainties calculated through the RSS method.

**Table 4:** Experimental and G3MP2B3-computed gas-phase enthalpies of formation of the benzoxazolones studied, at *T* = 298.15 K.

	$\Delta_{f\;}H_{m}^{{}^{\circ}}\left(g\right)$ $/\mathrm{kJ}\cdot {\mathrm{mol}}^{-1}$	
Compound	Experimental	G3MP2B3 ^1^
BOA	−219.0 ± 2.8 ^2^	−221.4 ± 3.4
3MBOA	−239.7 ± 3.2	−235.6 ± 1.0
6NBOA	−229.9 ± 3.7	−232.3 ± 4.7

Abbreviations: BOA, 2-benzoxazolinone; 3MBOA, 3-methyl-2-benzoxazolinone; 6NBOA, 6-nitro-2-benzoxazolinone. ^1^ More computational details are given in Tables S5–S7 of ESI; uncertainties are the expanded uncertainties of the mean (0.95 level of confidence), representing only an averaged result from the selected working reactions. ^2^ Reference [1].

**Table 5:** Standard (*p°* = 0.1 MPa) molar absolute entropies and standard molar entropies, enthalpies and Gibbs energies of formation of benzoxazolinones studied in the gaseous phase, at *T* = 298.15 K.

Compound	${S_{\;m}^{{}^{\circ}}}^{1}/J\cdot K^{-1}\cdot {\mathrm{mol}}^{-1}$	$\Delta_{f\;}S_{m}^{{}^{\circ}}/J\cdot K^{-1}\cdot {\mathrm{mol}}^{-1}$	$\Delta_{f\;}H_{m}^{{}^{\circ}}$ $/\mathrm{kJ}\cdot {\mathrm{mol}}^{-1}$	$\Delta_{f\;}G_{m\;}^{{}^{\circ}}$ $/\mathrm{kJ}\cdot {\mathrm{mol}}^{-1}$
BOA	346.84	−320.99	−219.0 ± 2.8 ^2^	−123.3 ± 2.8
3MBOA	379.52	−424.73	−239.7 ± 3.2	−113.1 ± 3.2
6NBOA	404.67	−498.77	−229.9 ± 3.7	−81.2 ± 3.7

Abbreviations: BOA, 2-benzoxazolinone; 3MBOA, 3-methyl-2-benzoxazolinone; 6NBOA, 6-nitro-2-benzoxazolinone. ^1^ Calculated at the B3LYP/6-31G(d) level of theory, using a vibrational frequency scaling factor of 1.0029 [15]. ^2^ Reference [1].

**Table 6:** Provenance and mass fraction purity of the compounds studied.

Compound	Molar Mass (g·mol^−1^)	CAS No.	Supplier	Purification Method	Final Mass Fraction Purity
3MBOA (C_8_H_7_NO_2_)	149.1463	21892-80-8	Sigma Aldrich, 98%	Sublimation ^1^	0.9999 ^2^
6NBOA (C_7_H_4_N_2_O_4_)	180.1174	4694-91-1	Alfa Aesar, 98%	Sublimation ^1^	0.9995 ^2^

Abbreviations: 3MBOA, 3-methyl-2-benzoxazolinone; 6NBOA, 6-nitro-2-benzoxazolinone. ^1^ Under reduced pressure. ^2^ Method of analysis: Gas–liquid chromatography.

## Data Availability

The data presented in this study are available in the Supplementary Materials.

## Supplementary Materials

The following are available online: Tables S1 and S2: Combustion calorimetry experiments of 3-methyl-2-benzoxazolinone and 6-nitro-2-benzoxazolinone; Tables S3 and S4: Values of standard molar heat capacities in the gaseous phase for the compounds studied; Figure S1: Optimized geometries of 2-benzoxazolinone derivatives; Tables S5–S7: Gas-phase hypothetical reactions and corresponding values for the enthalpies of reaction and formation, in the gaseous phase, at *T* = 298.15 K for 3-methyl-2-benzoxazolinone, 6-nitro-2-benzoxazolinone and 2-benzoxazolinone; Table S8: G3MP2B3-calculated absolute enthalpies, at *T* = 298.15 K, and experimental gas-phase values for all the molecules used; Table S9: Harmonic vibrational frequencies for 2-benzoxazolinone, 3-methyl-2-benzoxazolinone and 6-nitro-2-benzoxazolinone.
