# Supplementary material for: Experimental and Theoretical Investigation on the Thermochemistry of 3-Methyl-2-benzoxazolinone and 6-Nitro-2-benzoxazolinone
Source: Molecules. 2021 Dec 21;27(1):24. doi: 10.3390/molecules27010024 (PMC8746742; doi:10.3390/molecules27010024)
Supplement: Supplementary file 1 [file molecules-27-00024-s001.zip › molecules-1515495-supplementary.pdf]

SUPPLEMENTARY INFORMATION

Experimental and theoretical investigation on the thermochemistry of 3-methyl-2-benzoxazolinone and 6-nitro-2-benzoxazolinone

Ana L.R. Silva*, Vânia M. S. Costa, Maria D.M.C. Ribeiro da Silva

Research Center in Chemistry – CIQUP, Department of Chemistry and Biochemistry. Faculty of Sciences, University of Porto, Rua do Campo Alegre, 687, P-4169-007, Porto. Portugal

* Corresponding author:
analuisa.rs@fc.up.pt

This supplementary information includes:

- data of all the combustion calorimetry experiments of 3-methyl-2-benzoxazolinone and 6-nitro-2-benzoxazolinone (**Tables S1 and S2**);
- the values of standard molar heat capacities in the gaseous phase for the compounds studied (**Tables S3 and S4**);
- optimized geometries of 2-benzoxazolinone derivatives, calculated at the B3LYP/6-31G(d) level of theory (**Figure S1**);
- Gas-phase hypothetical reactions and corresponding values for the enthalpies of reaction and formation, in the gaseous phase, at *T* = 298.15 K for 3-methyl-2-benzoxazolinone, 6-nitro-2-benzoxazolinone and 2-benzoxazolinone (**Tables S5-S7**);
- G3MP2B3 calculated absolute enthalpies, at *T* = 298.15 K, and experimental gas-phase values for all the molecules used (**Table S8**);
- Harmonic vibrational frequencies for 2-benzoxazolinone, 3-methyl-2-benzoxazolinone and 6-nitro-2-benzoxazolinone (**Table S9**).

Acronyms used throughout this supplementary data:

3MBOA for 3-methyl-2-benzoxazolinone

6NBOA for 6-nitro-2(3*H*)benzoxazolone

Combustion Calorimetry:

- The calibration experiments were made in an oxygen atmosphere at p = 3.04 MPa, with 1.00 cm^3 of deionized water added to the bomb; the energy equivalent of the calorimeter was determined from the combustion of benzoic acid (NIST SRM 39j) having a massic energy of combustion, under standard bomb conditions, of –(26434 ± 3) $J{\cdot}g^{-1}$. From six calibration experiments ε_{cal} = (16002.6 ± 1.7) $J{\cdot}K^{-1}$, for an average mass of water added to the calorimeter of 3119.6 g; the mentioned uncertainty is the standard deviation of the mean.
- Samples of both compounds were burnt in pellet form, under oxygen at p = 3.04 MPa, with 1.00 cm^3 of deionised water added to the bomb; in the experiments of 6NBOA, a small amount of n-hexadecane (auxiliary of combustion) was used.
- n-Hexadecane (Aldrich, mass fraction > 0.999), stored under nitrogen was used as auxiliary combustion. The massic energy of combustion of the sample used, was determined in our laboratory as $\Delta_c u^\circ = -(47136.7 \pm 2.3)$ $J{\cdot}g^{-1}$.
- The cotton thread fuse (empirical formula: $CH_{1.686}O_{0.843}$) had a standard massic energy of combustion [S1], $\Delta_c u^\circ = -16240$ $J{\cdot}g^{-1}$;
- The ignition energy was determined from the change in potential difference on discharge of a 1400 μF condenser across a platinum wire.
- Pressure coefficient of specific energy $(\partial u/\partial p)_T = -0.2$ $J{\cdot}g^{-1}{\cdot}MPa^{-1}$ (typical value for organic compounds [S2]).
- The nitric acid formed in the calorimetric experiments was determined using Devarda's alloy method [S3] and the energy correction for the formation of 0.1 $mol{\cdot}dm^{-3}$ HNO_3(aq) [S4] from N_2 (g), O_2 (g) and H_2O (l) was based on −59.7 $kJ{\cdot}mol^{-1}$.
- Corrections for carbon formation relied on the standard massic energy of combustion of carbon [S5], $\Delta_c u^\circ = -32.76$ $kJ{\cdot}g^{-1}$.
- The calorimeter temperatures were measured to $\pm(1\times10^{-4})$ K, at time intervals of 10 s, with a quartz crystal thermometer (Hewlett-Packard HP 2804 A), interfaced to a PC; the ignition occurs at T = (298.150 ± 0.001) K.
- Specific density of the studied compounds at T = 298.15 K:
  ρ = 1.0831 $g{\cdot}cm^{-3}$ for 3MBOA [S6] and ρ = 1.58 $g{\cdot}cm^{-3}$ for 6NBOA [S7].
- The massic heat capacities, at T = 298.15 K, were calculated as 1.245 $J{\cdot}K^{-1}{\cdot}g^{-1}$ for 3MBOA and as 1.097 $J{\cdot}K^{-1}{\cdot}g^{-1}$ for 6NBOA, using Kopp's rule [S8].

Table S1. Standard (p^o = 0.1 MPa) mass energy of combustion of crystalline **3MBOA**, at T = 298.15 K.

$m(CO_2$, total) /g	1.44346	1.42080	1.77570	1.44647	1.52305	1.50243
m(cpd) /g	0.60941	0.59983	0.75045	0.61080	0.64339	0.63460
m(fuse) /g	0.00301	0.00298	0.00257	0.00284	0.00262	0.00270
T_i /K	298.1504	298.1514	298.1592	298.1524	298.1512	298.1507
T_f /K	299.2100	299.1984	299.4260	299.2116	299.2640	299.2570
ΔT_{ad} /K	0.98107	0.96581	1.20624	0.98357	1.03534	1.02109
ε_i /(J·K^{-1})	15.27	15.26	15.43	15.27	15.31	15.30
ε_f /(J·K^{-1})	15.67	15.58	15.97	15.65	15.66	15.68
$-\Delta U$(IBP) /J	15713.90	15469.82	19321.53	15754.06	16583.72	16355.35
$\Delta U(HNO_3)$ /J	27.22	33.73	29.25	28.84	35.58	31.46
ΔU(ign) /J	1.15	0.70	0.71	1.02	0.63	0.76
ΔU_Σ /J	12.00	11.76	15.07	12.02	12.69	12.52
$-\Delta U$(fuse) /J	48.88	48.40	41.74	46.12	42.55	43.85
$-\Delta_c u^o$ /(J·g^{-1})	25640.85	25633.74	25631.77	25649.99	25634.31	25634.21

% CO_2 = (100.008 ± 0.001)[a]

$-\langle\Delta_c u^\circ\rangle$ = (25637.48 ± 6.86) J·g^{-1} [a]

$m(CO_2$. total) is the total mass of CO_2 recovered in the experiment; m(cpd) is the mass of compound burnt in each experiment; m(fuse) is the mass of fuse (cotton) used in each experiment; T_i is the initial temperature rise; T_f is the final temperature rise; ΔT_{ad} is the corrected temperature rise; ε_i and ε_f are the energy equivalents of contents in the initial and final states, respectively; ΔU(IBP) is the energy change for the isothermal combustion reaction under actual bomb conditions and includes ΔU(ign); $\Delta U(HNO_3)$ is the energy correction for the nitric acid formation; ΔU(ign) is the electrical energy for ignition; ΔU_Σ is the standard state correction; ΔU(fuse) is the energy of combustion of the fuse (cotton); $\Delta_c u^\circ$ is the standard massic energy of combustion. [a] Mean value and standard deviation of the six experiments.

Table S2. Standard (p^o = 0.1 MPa) mass energy of combustion of crystalline **6NBOA**, at T = 298.15 K.

$m(CO_2, total)$ /g	1.06543	1.31205	1.02819	1.28084	1.26352	1.26658
m(cpd) /g	0.62026	0.51303	0.52849	0.52977	0.51087	0.52546
m(fuse) /g	0.00281	0.00265	0.00301	0.00302	0.00291	0.0019
m(n-hexadec.) /g	—	0.13837	0.03899	0.11927	0.12417	0.11731
T_i /K	298.1506	298.1514	298.1506	298.1509	298.1513	298.1506
T_f /K	298.8918	299.1725	298.9076	299.1394	299.1308	299.125
ΔT_{ad} /K	0.64406	0.94067	0.66342	0.90169	0.89586	0.89061
ε_i /(J·K^{-1})	15.20	15.39	14.91	15.37	15.36	15.08
ε_f /(J·K^{-1})	15.23	15.74	15.00	15.68	15.69	15.38
$-\Delta U$(IBP) /J	10315.53	15067.22	10625.74	14443.04	14349.52	14264.63
$\Delta U(HNO_3)$ /J	27.34	35.58	29.31	34.15	32.48	33.49
ΔU(ign) /J	0.91	0.75	0.66	0.48	0.63	1.14
ΔU_Σ /J	12.32	11.79	10.88	11.91	11.57	11.78
ΔU(carb.) /J	—	—	16.50	9.24	9.90	—
$-\Delta U$(n-hexadec.) /J	—	6522.37	1837.78	5621.89	5853.14	5529.38
$-\Delta U$(fuse) /J	45.63	43.04	48.88	49.04	47.26	30.86
$-\Delta_c u^o$ /(J·g^{-1})	16493.48	16479.43	16491.11	16488.84	16471.83	16479.13

% CO_2 = (100.06 ± 0.02) [b]

$-\langle\Delta_c u^\circ\rangle$ = (16483.97 ± 3.45) J·g^{-1} [b]

$m(CO_2$. total) is the total mass of CO_2 recovered in the experiment; m(cpd) is the mass of compound burnt in each experiment; m(fuse) is the mass of fuse (cotton) used in each experiment; m(n-hexadec.) is the mass of n-hexadecane used in each experiment; T_i and T_f are the initial and final temperatures rise, respectively; ΔT_{ad} is the corrected temperature rise; ε_i and ε_f are the energy equivalents of contents in the initial and final states, respectively; ΔU(IBP) is the energy change for the isothermal combustion reaction under actual bomb conditions and includes ΔU(ign); $\Delta U(HNO_3)$ is the energy correction for the nitric acid formation; ΔU(ign) is the electrical energy for ignition; ΔU_Σ is the standard state correction; ΔU(carb.) is the correction energy for carbon soot formation; ΔU(n-hexadec.) is the energy of combustion of the n-hexadecane; ΔU(fuse) is the energy of combustion of the fuse (cotton); $\Delta_c u^\circ$ is the standard massic energy of combustion.[a] Mean value and standard deviation of the mean.

Table S3. Standard (p^o = 0.1 MPa) molar heat capacities in the gaseous phase for **3MBOA .**

T / K	$C^{\circ}_{p,\,m}$ (g) / $(J{\cdot}K^{-1}{\cdot}mol^{-1})$
150	84.72
200	106.70
250	129.97
298.15	152.58
300	153.44
350	176.09
400	197.25
450	216.59
500	234.06
550	249.73

$$C^{\circ}_{p,\,m}(g) = -6.769\times10^{-7}\ T^3 + 4.907\times10^{-4}\ T^2 + 3.449\times10^{-1}\ T + 23.94$$

Table S4. Standard (p^o = 0.1 MPa) molar heat capacities in the gaseous phase for **6NBOA .**

T / K	$C^{\circ}_{p,\,m}$ (g) / $(J{\cdot}K^{-1}{\cdot}mol^{-1})$
300	167.55
350	190.26
400	210.83
450	229.21
500	245.46
550	259.79
600	272.42
650	283.57
700	293.44

$$C^{\circ}_{p,\,m}(g) = 2.249\times10^{-7}\ T^3 - 7.113\times10^{-4}\ T^2 + 8.484\times10^{-1}\ T - 29.07$$

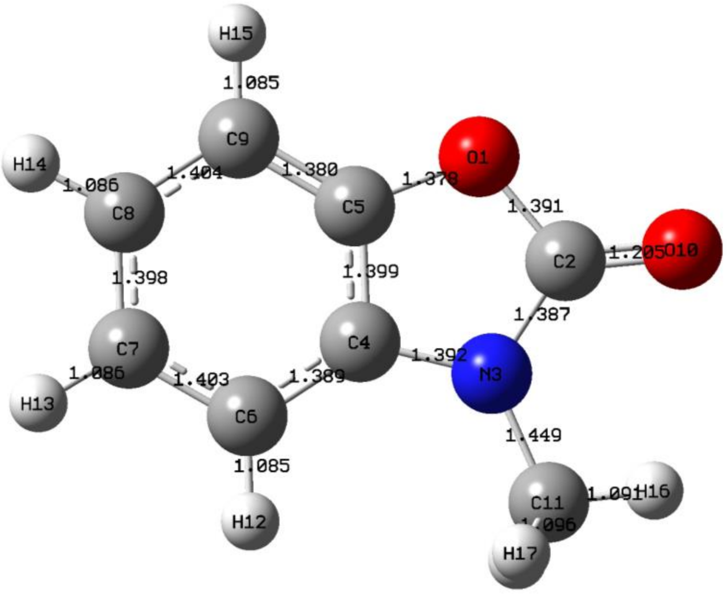

3MBOA

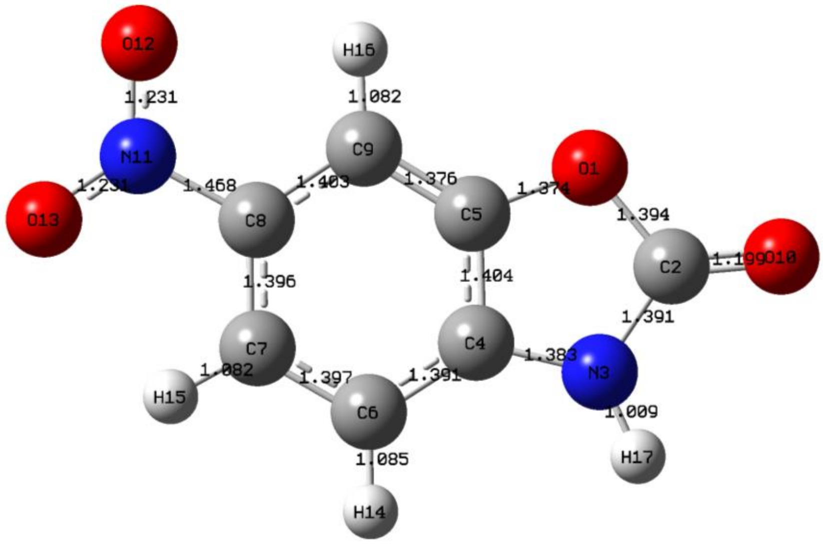

6NBOA

Figure S1. Molecular structure of the compounds studied in the gaseous phase, optimized by B3LYP/6-31G(d) level of theory, with the corresponding calculated bond distances (10^{-10} m).

Table S5. Gas-phase hypothetical reactions for **3MBOA** and corresponding values for the enthalpies of reaction, $\Delta_r H_m^\circ$, and formation, $\Delta_f H_m^\circ$, in the gaseous phase, at T = 298.15 K (values in kJ·mol^{-1}).

Gas-phase hypothetical reactions	Eq.	$\Delta_r H_m^o$	$\Delta_f H_m^o$
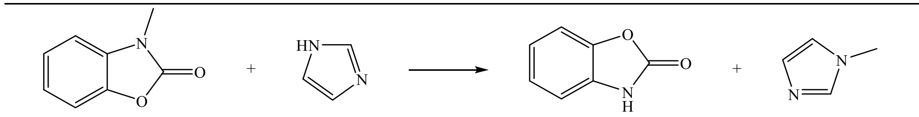 3-methyl-2-benzoxazolinone + imidazole → 2-benzoxazolinone + 1-methylimidazole	I	10.10	–236.3
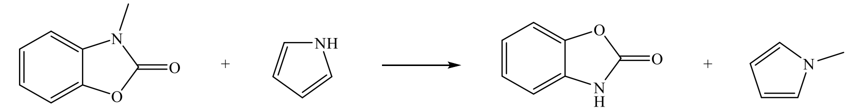 3-methyl-2-benzoxazolinone + pyrrole → 2-benzoxazolinone + 1-methylpyrrole	II	11.46	–235.8
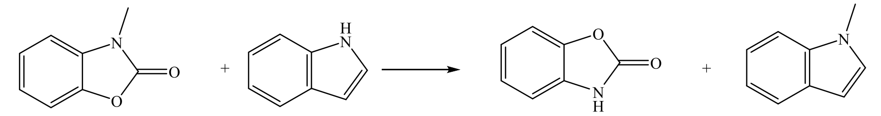 3-methyl-2-benzoxazolinone + indole → 2-benzoxazolinone + 1-methylindole	III	7.83	–235.3
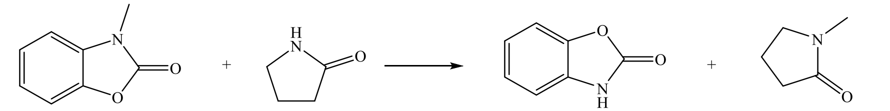 3-methyl-2-benzoxazolinone + 2-pyrrolidinone → 2-benzoxazolinone + 1-methyl-2-pyrrolidinone	IV	3.78	–236.2
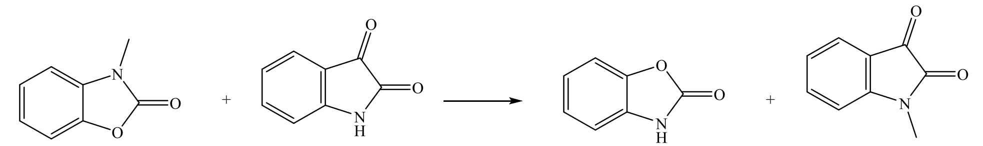 3-methyl-2-benzoxazolinone + 2,3-dioxoindole → 2-benzoxazolinone + 1-methyl-2,3-dioxoindole	V	–0.28	–234.7
		Mean Value	–235.6 ± 1.0 [1]

[1] The assigned uncertainty is an expanded uncertainty of the mean using an appropriate coverage factor, k = 2.776, to provide a level of confidence of 0.95.

Table S6. Gas-phase hypothetical reactions for **6NBOA** and corresponding values for the enthalpies of reaction, $\Delta_r H_m^\circ$, and formation, $\Delta_f H_m^\circ$, in the gaseous phase, at T = 298.15 K (values in kJ·mol^{-1}) .

Gas-phase hypothetical reactions	Eq.	$\Delta_r H_m^o$	$\Delta_f H_m^o$
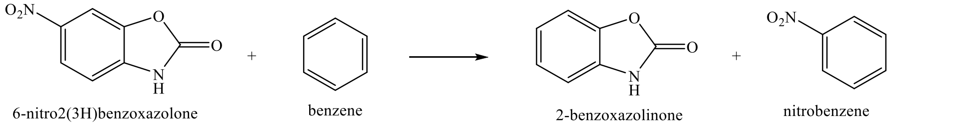	I	–4.42	–229.7
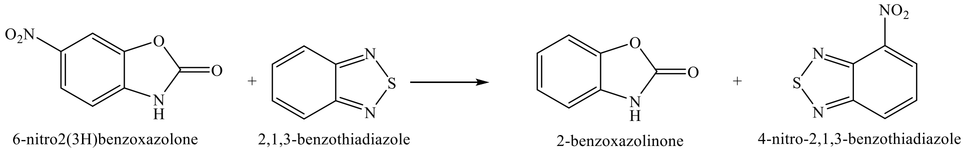	II	23.92	–235.7
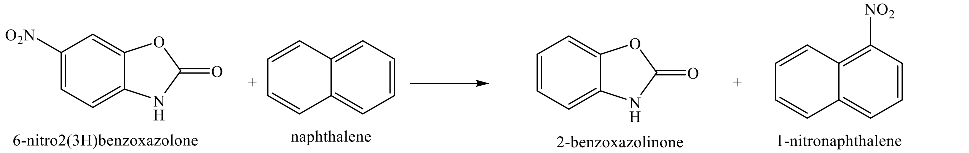	III	10.43	–234.7
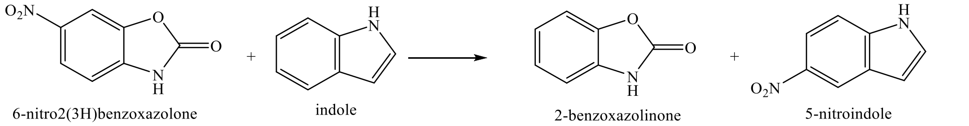	IV	–11.84	–234.6
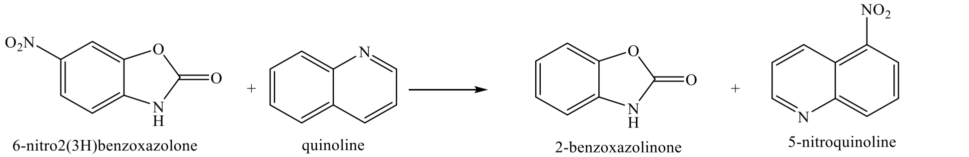	V	10.85	–226.9
		Mean Value	–232.3 ± 4.7 [1]

[1] The assigned uncertainty is an expanded uncertainty of the mean using an appropriate coverage factor, k = 2.776, to provide a level of confidence of 0.95.

Table S7. Gas-phase hypothetical reactions for **BOA** and corresponding values for the enthalpies of reaction, $\Delta_r H^\circ_m$, and formation, $\Delta_f H^\circ_m$, in the gaseous phase, at T = 298.15 K (values in kJ·mol^{-1}) .

Gas-phase hypothetical reactions	Eq.	$\Delta_r H^o_m$	$\Delta_f H^o_m$
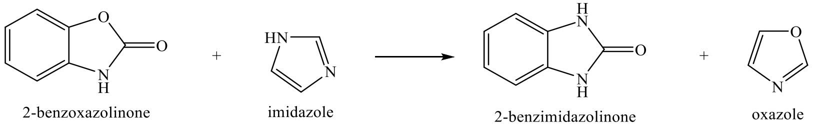	I	7.33	–219.6
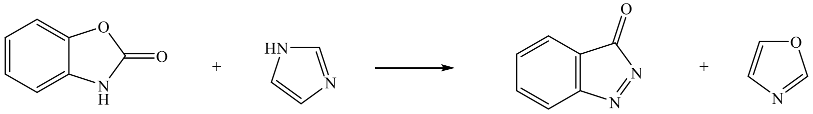	II	144.40	–222.8
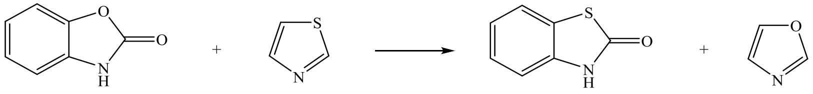	III	18.20	–219.4
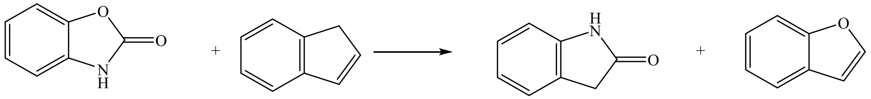	IV	7.03	–223.6
		Mean Value	–221.4 ± 3.4 [1]

[1] The assigned uncertainty is an expanded uncertainty of the mean using an appropriate coverage factor, k = 3.182, to provide a level of confidence of 0.95.

Table S8. G3MP2B3 absolute enthalpies (in *Hartrees**) and experimental enthalpies of formation (in kJ·mol^{-1}), in the gaseous phase, at T = 298.15 K, for the studied compounds and the auxiliary molecules used in the gas-phase working reactions.

Compounds studied / auxiliary molecules	G3MP2B3 absolute enthalpies	Experimental enthalpies of formation (298.15 K)
3-Methyl-2-benzoxazolinone	-513.535373	(–239.7 ± 3.2)[1]
6-Nitro-2-benzoxazolinone	-678.598843	(–229.9 ± 3.7)[1]
1*H*-imidazole	-225.873394	(132.9 ± 0.6) [S9]
1-Methylimidazole	-265.103073	(125.7 ± 1.1) [S10]
2-Benzoxazolinone	-474.301846	(–219.0 ± 2.8) [S9]
Indole	-363.214962	(164.3 ± 1.3) [S9]
1-Methylindole	-402.445506	(155.8 ± 2.8) [S9]
Pyrrole	-209.822531	(108.4 ± 0.6) [S9]
1-Methylpyrrole	-249.051695	(103.1 ± 0.5) [S9]
2-Pyrrolidinone	-286.188826	(–197.4 ± 3.1) [S9]
1-Methyl-1-pyrrolidinone	-325.420913	(–210.9 ± 0.6) [S9]
2,3-Dioxoindole	-512.331688	(–133.0 ± 5.6) [S9]
1-Methyl-2,3-dioxoindole	-551.565323	(–148.6 ± 4.4) [S9]
Benzene	-231.835164	(82.6 ± 0.7) [S9]
Nitrobenzene	-436.133844	(67.5 ± 0.5) [S9]
2,1,3-Benzothiadiazole	-737.775911	(276.5 ± 2.5) [S11]
4-Nitro-2,1,3-benzothiadiazole	-942.063797	(283.7 ± 4.9) [S12]
Naphthalene	-385.223727	(150.3 ± 1.4) [S9]
1-Nitronaphthalene	-589.516753	(145.0 ± 1.9) [S13]
5-Nitroindole	-567.51647	(136.9 ± 2.1) [S14]
Quinoline	-401.263696	(200.5 ± 0.9) [S15]
5-Nitroquinoline	-605.556561	(203.4 ± 2.6) [S16]
Oxazole	-245.727307	(–15.5 ± 0.5) [S9]
Thiazole	-568.350825	[151.5][2]
3-Indazolinone	-454.392934	(70.0 ± 2.2) [S9]
Indene	-347.156638	(163.4 ± 2.0) [S9]
Oxindole	-438.385869	(–66.8 ± 3.2) [S9]
2-Benzimidazolinone	-454.445141	(–63.9 ± 2.9) [S9]
2-Benzothiazolinone	-796.918431	(–34.2 ± 2.7) [S9]

[1] This work. [2] Value estimated at the G3(MP2)//B3LYP level of theory.
* 1 Hartree = 2625.5 kJ·mol^{-1}

Table S9. Harmonic vibrational frequencies (scaling vibrational frequencies by 1.0029 [S17]) **.**

2-benzoxazolinone		3-methyl-2- benzoxazolinone		6-nitro-2-benzoxazolinone	
126.0	1279.7	121.9	1120.4	61.8	964
243.3	1295.8	129.4	1153.2	92.5	966.5
318.2	1313.2	138.3	1172.2	164.4	1080
320.7	1368.9	241.8	1193.7	188.6	1132.4
419.8	1446.3	263.5	1255.8	270.5	1165.5
463.3	1530.8	334.8	1302.2	335.6	1271.4
511.5	1532.6	341.6	1347.8	347.3	1286.2
577.1	1681.4	439.1	1402.4	354.6	1306.3
580.6	1682.9	507.7	1436.9	433.1	1363.3
629.4	1918.2	569.5	1478.8	470.1	1395.6
708.5	3206.4	580.7	1522	525.3	1462.2
733.3	3218.7	636.2	1525.4	562.7	1501.6
744.3	3228.0	692.9	1539.4	579.5	1533.4
759.5	3241.5	729.5	1546.9	597.8	1637.7
860.2	3683.8	740.1	1673.3	698.8	1677.9
870.6		754.1	1684.3	717.6	1686.5
903.0		759.6	1895.5	723.8	1930.9
922.9		859.3	3064	744.5	3232.3
940.1		886	3122.1	752.9	3268.3
968.9		921.1	3185.9	838.1	3280.6
1043.4		957.5	3205.7	841.2	3682.8
1112.1		968.9	3219	885.1	
1164.2		1043.2	3228.5	913.2	
1193.4		1070.6	3240.6	935.4	

REFERENCES

[S1] Coops, J.; Jessup, R.S.; van Nes, K. In *Experimental Thermochemistry*. Rossini, F.D. Ed. Vol. 1, Interscience, New York, 1956, Chapter 3.

[S2] Washburn, E.W. Standard states for bomb calorimetry. *J. Res. Nat. Bur. Stand*. **1933**, *10*, 525-558.

[S3] Vogel, A.I. *Quantitative Inorganic Analysis*; Longman: London, 1978.

[S4] Wagman, D.D.; Evans, W.H.; Parker, V.B.; Schumm, R.H.; Halow, I.; Bailey, S.M.; Churney, K.L.; Nuttall, R.L. J. Phys. Chem. Ref. Data **1982**, 11(suppl. 2).

[S5] Medvedev, V.A.; Cox, J.D.; Wagman, D.D. (Eds). (1989) CODATA key values for thermodynamics. New York: Hemisphere Publishing Corporation.

[S6] Yaws, C.L. Thermophysical Properties of Chemicals and Hydrocarbons, 2008, William Andrew Inc., USA

[S7] https://www.chemicalbook.com/ChemicalProductProperty_US_CB6747758.aspx

[S8] Hurst, J.E.; Harrison, B.K. Estimation of liquid and solid heat capacities using a modified Kopp's rule. *Chem. Eng. Comm*. **1992**, *112*, 21-30.

[S9] Pedley, J.P. Thermochemical data and structures of organic compounds. Thermodynamics Research Centre, College Station, TX, 1994.

[S10] Verevkin, S.P.; Zaitsau, D.H.; Emel'yanenko, V.N.; Paulechka, Y.U.; Blokhin, A.V.; Bazyleva, A.B.; Kabo, G.J. Thermodynamics of ionic liquids precursors: 1-methylimidazole. *J. Phys. Chem. B* **2011**, *115*, 15, 4404–4411.

[S11] Miranda, M.S.; Matos, M.A.R.; Morais, V.M.; Liebman, J.F. (2012). 2,1,3-Benzothiadiazole: Study of its structure, energetics and aromaticity. *J. Chem. Thermodyn*. **2012**, *50*, 30-36.

[S12] Ribeiro da Silva, M.D.M.C.; Freitas V.L.S.; Vieira M.A.A.; Sottomayor, M.J.; Acree Jr. W.E. Energetic and structural properties of 4-nitro-2,1,3-benzothiadiazole. J. Chem. Thermodyn. 2012, 49, 146–153.

[S13] Ribeiro da Silva, M.A.V.; Amaral, L.M.P.F.; Santos, A.F.L.O.M.; Gomes, J.R.B. Thermochemistry of nitronaphthalenes and nitroanthracenes. *J. Chem. Thermodyn.* **2006**, *38*, 748-755.

[S14] Ribeiro da Silva, M.A.V.; Cabral, J. I.T.A. Experimental study on the thermochemistry of 5-nitroindole and 5-nitroindoline. *J. Chem. Thermodyn*. **2009**, *41*, 355-360.

[S15] Steele, W.V.; Archer, D.G.; Chirico, R.D.; Collier, W.B.; Hossenlopp, I.A.; Nguyen, A.; Smith, N.K.; Gammon, B.E. *J. Chem. Thermodyn.* **1988**, *20*, 1233-1264.

[S16] Ribeiro da Silva, M.A.V.; Matos, M.A.R.; Amaral, L.M.P.F. Enthalpies of combustion, vapour pressures, and enthalpies of sublimation of 5-, 6-, and 8-nitroquinoline and 8-nitroquinaldine. *J. Chem. Thermodyn.* **1997**, *29*, 298-303.

[S17] Merrick, J.P., Moran, D.; Radom, L. An Evaluation of Harmonic Vibrational Frequency Scale Factors, *J. Phys. Chem. A* **2007**, *111*, 11683–11700.
